# The correlates of benefit from neoadjuvant chemotherapy before surgery in non-small-cell lung cancer: a metaregression analysis

**DOI:** 10.1186/1477-7819-10-161

**Published:** 2012-08-09

**Authors:** Hakan Bozcuk, Huseyin Abali, Senol Coskun

**Affiliations:** 1 Akdeniz University Hospital, Dept. of Medical Oncology, Antalya, Turkey; 2 Adana Başkent Hospital, Dept. of Medical Oncology, Adana, Turkey

**Keywords:** Meta-analysis, Metaregression, Mortality, Neoadjuvant chemotherapy, Non-small-cell lung cancer

## Abstract

**Background:**

Although neoadjuvant chemotherapy (NCT) is widely used, it is not clear which subgroup of locally advanced non-small-cell lung cancer (NSCLC) patients should be treated with this approach, and if a particular benefit associated with NCT exists. In this study, we aimed to investigate the potential correlates of benefit from NCT in patients with NSCLC.

**Methods:**

All randomized clinical trials (RCTs) utilizing a NCT arm (without radiotherapy) versus a control arm before surgery were included for metaregression analysis. All regression analyses were weighed for trial size. Separate analyses were conducted for trials recruiting patients with different stages of disease. Previously published measures of treatment efficacy were used for the purpose of this study, regardless of being published in full text or abstract form.

**Results:**

A total of 14 RCTs, consisting of 3,615 patients, were selected. Histology, stage, various characteristics of the NCT protocol, and different trial features including trial quality score were not associated with the benefit of NCT. However, in trials of stage 3 disease only, there was a greater benefit in terms of reduction in mortality from NCT, if protocols with three chemotherapeutics were used (B = −0.18, t = −5.25, *P* = 0.006).

**Conclusions:**

We think that patients with stage 3 NSCLC are served better with NCT before surgery if protocols with three chemotherapy agents or equally effective combinations are used. In addition, the effect of neoadjuvant chemotherapy is consistent with regard to disease and patient characteristics. This finding should be tested in future RCTs or individual patient data meta-analyses.

## Background

Locally advanced cases of non-small-cell lung cancer (NSCLC) constitute nearly a fifth of all NSCLC cases [1,2]. Different standards exist for the treatment of locally advanced NSCLC, partially owing to the heterogeneous nature of this disease, and lack of consistent data regarding treatment outcomes [2,3].

Although chemoradiotherapy and neoadjuvant chemotherapy (NCT) followed by surgery are two widely utilized therapeutic approaches, NCT is still considered as experimental, primarily because previous meta-analyses have been inconclusive [2-7]. The last meta-analysis in particular shows the benefit of NCT, but fails to point out any special feature that is linked with the magnitude of efficacy of NCT [8]. Therefore, from a practical point of view, the important questions of which patients should be treated with NCT, and whether there is any superior type of NCT, still remain to be solved.

Related to these uncertainties regarding NCT, we aimed to formulate hypotheses by looking into various factors in previous randomized controlled trials (RCTs) of NSCLC, which could be linked with the magnitude of benefit from NCT, and thus, decided to conduct a metaregression analysis to explore these factors.

## Materials and methods

### Inclusion and exclusion of trials

All RCTs reported after 1966 with an NCT arm versus a control arm (no NCT arm) before surgery were included in this analysis. Two reviewers (HB, HA) selected RCTs and extracted the data. All stages and full text or abstract formats in English language literature only were allowed. References of key articles were also referred to. Hazard ratio (HR) was selected as summary data for treatment efficiency for mortality, and used as given in the last full text meta-analysis [7], or in the original publication of trials.

Non-randomized trials, as well as those with the inclusion of radiotherapy before surgery (that is, induction chemoradiotherapy trials) were excluded from the analysis.

### Metaregression analysis

Hazard ratios for mortality for individual trials were log transformed prior to being used as independent variables in the linear regression analysis. All analyses were weighed for the square root of individual trial size, as a conservative measure of the (trial size adjusted) effect of individual RCTs. However, for exploratory purposes, we also tested the effect of trial size on the magnitude of the associated hazard ratio of individual RCTs, and in these analyses only, weighting for the square root of trial size was not carried out. All trials, and also those with stage 3 only, and mixed stage trials (stage 1 to 3), were separately analyzed. A *P* value <0.05 was considered to be significant.

A trial quality score was constructed using a scale from 0 to 3, depending on three features; randomization method (details given vs not given), stratification criteria (reported vs not reported), and trial type (reported in full text or abstract). So, 0 or 1 point for each trial feature was totaled for the final trial quality score. We also drew simple regression lines to illustrate the relationship between mortality and its predictors in clinically significant subgroups as shown in the literature [9].

## Results

A total of 3,615 patients were represented in 14 RCTs [10-26]. The median number of cases in each trial was 240.5 (26 to 624). Publication years varied between 1990 and 2010, and the median fraction of patients with squamous cell cancer was 51.5%. A median of three cycles of NCT (1 to 3) was used and the median trial quality score was 1 (0 to 3). Six of the trials (42.9% of all trials) addressed patients with stage 3 only disease, whereas eight trials (57.1%) included patients with mixed stage (1 to 3) NSCLC. See Table 1 for individual RCT characteristics.

**Table 1:** Characteristics, methodological quality, treatment employed and outcome of the trials

**Trial**	**Reference**	**Recruitment period**	**Publication year**	**Trial size**	**Origin of study**	**Randomization method**	**Stratification criteria**	**Trial type**	**Stage**	**Fraction with squamous cell (%)**	**Details of neoadjuvant treatment**	**HR**^**a**^**(95% CI)**
Dautzenberg *et al.*	10	1985 to 1987	1990	26	European	No details given	Not reported	Full text	Stage 1 to 3	81	VCP × 2	1.10 (0.41 to 2.93)
Roth *et al.*	13,14	1987 to 1993	1998	60	US	No details given	Not reported	Full text	Stage 3 only	37	CEP × 3	0.56 (0.31 to 1.01)
Rosell *et al.*	11, 12	1989 to 1991	1999	60	European	Details given	Not reported	Full text	Stage 3 only	70	MIP × 3	0.50 (0.30 to 0.85)
Zhou *et al.*	21	1990 to 2001	2001	624	Asian	Details given	Not reported	Full text	Stage 3 only	51	BAI/MVP/CAP/EP/VIP/GP/NP/PC/TN × 2	0.87 (0.71 to 1.07)
De Pierre *et al.*	15	1991 to 1997	2002	355	European	No details given	Reported	Full text	Stage 1 to 3	74	MIP × 2	0.83 (0.64 to 1.07)
JCOG	16	1993 to 1998	2003	62	Asian	No details given	Reported	Full text	Stage 3 only	24	VP × 3	1.19 (0.69 to 2.05)
Li *et al.*	24	1990 to 1995	2003	137	Asian	No details given	Not reported	Full text	Stage 3 only	80	CAP/EP × 1	0.68 (0.46 to 1.00)
Liao *et al.*	22	1995 to 1997	2003	211	Asian	Details given	Reported	Full text	Stage 1 to 3	-	MVP/MAP × 2	1.06 (0.76 to 1.48)
Yao *et al.*	23	1990 to 2002	2004	456	Asian	Details given	Not reported	Full text	Stage 3 only	55	GP/NP/MVP/EP × 2	0.83 (0.67 to 1.03)
Sorensen *et al.*	17	1998 to 2004	2005	90	US	No details given	Not reported	Abstract	Stage 1 to 3	-	TP × 3	0.91 (0.55 to 1.50)
s9900	18,19	1999 to 2004	2006	336	European	No details given	Not reported	Full text	Stage 1 to 3	38	PC × 3	0.79 (0.60 to 1.06)
MRCLU22	6	1997 to 2005	2007	519	European	No details given	Not reported	Full text	Stage 1 to 3	49	MVP/MIP/NP/PC/DC/GP × 3	1.02 (0.80 to 1.31)
Ch.E.S.T	20, 25	2000 to 2004	2008	270	European	No details given	Not reported	Abstract	Stage 1 to 3	41	GP × 3	0.63 (0.46 to 0.87)
Felip *et al.*	26	2000 to 2007	2010	409^b^	European	No details given	Not reported	Full text	Stage 1 to 3	52	PC × 3	0.96 (0.84 to 1.10)

^a^Hazard ratio for mortality.

^b^Number of cases in surgery only, and neoadjuvant chemotherapy arms; excludes patients in the adjuvant chemotherapy arm.

BAI = bronchial artery infusion; CAP = cyclophosphamide, adriamycin, cisplatin; CEP = etoposide, cyclophosphamide, cisplatin; DC = docetaxel, carboplatin; EP = etoposide, cisplatin; GP = gemcitabine, cisplatin; MAP = mitomycin, adriamycin, cisplatin; MIP = mitomycin, ifosfamide, cisplatin; MVP = mitomycin, vindesine, cisplatin; NP = navelbine, cisplatin; PC = paclitaxel, carboplatin; TN = paclitaxel, navelbine; VCP = vindesine, cyclophosphamide, cisplatin; VIP = vindesine, ifosfamide, cisplatin; VP = vindesine, cisplatin.

None of the factors evaluated in the regression analysis were linked with the efficacy of NCT. These factors were histology, stage, type of platinum drug used, generation and number of drugs in the protocol, number of cycles administered before surgery, trial size, publication year, origin of study and trial quality score. Only stage of disease interacted with the number of chemotherapy drugs used to define the magnitude of NCT benefit. In stage 3 patients, reduced risk of mortality was associated with the utilization of more frequent usage of three drug regimes (B = −0.18, t = −5.25, *P* = 0.006). Table 2 shows details of the regression analysis. The R^2^ value for the association of log transformed hazard ratios for mortality with number of drugs in the protocol was 0.92 in trials of stage 3 disease, and 0.27 in trials of mixed stages (stages 1 to 3).

**Table 2:** Factors associated with benefit from neoadjuvant chemotherapy in non-small-cell lung cancer

**Factors**	**All trials**		**Stage 1 to 3 (mixed-stage trials)**		**Stage 3**	
	**B (95% CI)**^**a**^	***P*value**	**B (95% CI)**	***P*value**	**B (95% CI)**	***P*value**
Disease characteristics						
Histology (squamous cell fraction)	−0.00 (−0.01 to 0.00)	0.600	0.00 (−0.01 to 0.01)	0.536	−0.00 (−0.01 to 0.00)	0.261
Stage (1 to 3 vs 3 only)	−0.06 (−0.17 to 0.05)	0.256	NR^b^	NR^b^	NR^b^	NR^b^
Treatment characteristics						
Platinum type (carboplatin vs cisplatin based)	−0.07 (−0.15 to 0.04)	0.205	−0.05 (−0.18 to 0.09)	0.411	NR^c^	NR^c^
Generation of drugs (older vs third-generation agent protocol)	−0.03 (−0.09 to 0.14)	0.639	−0.04 (−0.19 to 0.11)	0.572	−0.09 (−0.16 to 0.34)	0.373
Number of drugs (2 vs 2 to 3 vs 3 drugs)	−0.01 (−0.09 to 0.06)	0.695	−0.03 (−0.04 to 0.11)	0.303	−0.18 (−0.27 to −0.08)	**0.006**
Number of chemotherapy cycles	0.00 (−0.09 to 0.10)	0.955	−0.04 (−0.19 to 0.11)	0.572	−0.01 (−0.23 to 0.21)	0.871
Trial characteristics						
Trial size^d^	0.00 (−0.01 to 0.01)	0.569	−0.00 (−0.02 to 0.01)	0.530	0.01 (−0.02 to 0.03)	0.514
Publication year	0.01 (−0.01 to 0.02)	0.605	−0.00 (−0.02 to 0.01)	0.535	0.03 (−0.03 to 0.09)	0.215
Origin of study (European vs Asian vs US)	−0.01 (−0.10 to 0.08)	0.885	0.03 (−0.09 to 0.15)	0.566	0.03 (−0.03 to 0.34)	0.841
Trial quality score^e^	0.03 (−0.04 to 0.09)	0.373	0.04 (−0.03 to 0.10)	0.186	0.12 (−0.16 to 0.40)	0.300

^a^Regression coefficient and 95% confidence interval.

^b^Not relevant.

^c^Analysis not performed due to insufficient number of trials in that subgroup.

^d^Square root transformation. For analyses for trial size, analyses have not been weighed for trial size.

^e^Over a scale from 0 to 3, depending on randomization method (details given vs not given), stratification criteria (reported vs not reported) and trial type (reported in full text or abstract).

When only trials recruiting solely stage 3 patients were considered, patients who received three drugs obtained a 35% reduction in hazard ratio for mortality compared to those that received two to three drugs (hazard ratio = 0.53 in three drug trials, versus 0.81 in two to three drug, heterogeneous treatment design trials). In the only trial utilizing two drugs, neoadjuvant chemotherapy was associated with a 19% increased risk of mortality over no neoadjuvant chemotherapy (hazard ratio = 1.19).

**Figure 1:**
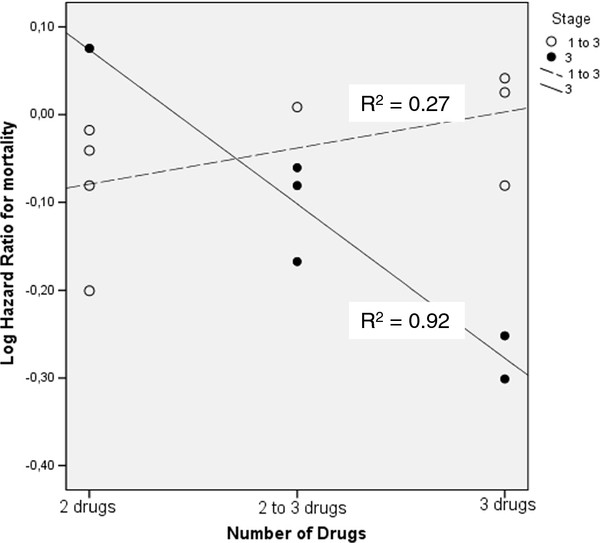
**Number of drugs in the neoadjuvant protocol and risk of death with respect to stage of non-small-cell lung cancer.** This figure shows the activity of different numbers of chemotherapy drugs in the neoadjuvant chemotherapy protocol in different stages of disease.

The association with NCT benefit of protocols with two or three chemotherapeutics in stage 3 or other stages is represented in Figure 1. As only a minority of the trials reported data on the toxicity of NCT, we did not conduct a separate analysis to define correlates of toxicity.

## Discussion

We show for the first time that if NCT is to be used, a higher number of chemotherapy drugs in the treatment protocol may be associated with decreased mortality in stage 3 NSCLC. However, in contrast, the most recent individual patient data (IPD) meta-analysis failed to demonstrate any factor that was associated with NCT benefit in NSCLC [8]. It would be worthwhile to test the validity of our hypothesis in this IPD meta-analysis dataset. Of note, in the literature, a large NCT RCT with three drug protocols containing third-generation agents with at least three cycles of treatment and conducted solely in stage 3 NSCLC patients is lacking. To have such a RCT would obviously provide great insight into the current management of stage 3 disease in NSCLC.

Third-generation three-drug protocols have previously been tried in small trials in the setting of stage 3 NSCLC [27,28]. Gemcitabine, vinorelbine, cisplatin (GVP) particularly, appear to be promising among these regimens, with a pathological complete response rate of 25% [27]. Therefore, future RCTs using these three-drug protocols for NCT are warranted. In addition, proper staging in these future trials of distant disease as well as of mediastinum before and after NCT, with positron emission tomography-computed tomography (PET-CT), other imaging modalities, and invasive and non-invasive means of pathological mediastinal evaluation better helping to properly quantify the amount of benefit from these protocols.

Obviously, predictive biomarker studies integrated within NCT trials are also of great importance, as some potential predictive markers have been shown to be of use in metastatic NSCLC. ERCC1 and RRM1 are two such markers that have been linked with the benefit of cisplatin and gemcitabin based protocols, respectively [29,30]. We think integrating different biomarkers and genomic profiling in treatment protocols of stage 3 NSCLC is also very important, since the disease biology and available treatment options in this setting are heterogeneous and each patient should be carefully evaluated on an individual basis to tailor the best treatment.

A limitation of this study is the long time period from which the trials included in the study were collected (from 1990 to 2010). Staging techniques and treatment protocols may have changed during this period of time; therefore one has to be aware of this fact when making comparisons between different studies. Particularly, integration of PET/CT into staging investigations has considerably changed routine clinical practice for patients with NSCLC, and future neoadjuvant studies have to make good use of invasive and non-invasive methods of staging, accordingly.

In a previous study, our group had investigated the correlates of benefit from neoadjuvant chemotherapy before radiotherapy in NSCLC; our current attempt extends this effort to the benefit of NCT before surgery [31]. Because personalizing treatment in stage 3 disease would also require various predictive markers in the context of different treatment settings, just as in stage 4 disease, predictive markers that are of proven benefit will be readily welcomed by thoracic oncologists.

It is interesting that in our analysis, the benefit from NCT was not different with respect to the stage of disease. In addition, an indirect methodology meta-analysis had shown that NCT was similar to adjuvant chemotherapy in terms of survival benefit provided [32]. These findings taken together suggest that when chemotherapy is indicated in stage 2 or 3 disease, NCT or adjuvant chemotherapy are reasonable options in suitable patients. It may be NCT is more beneficial for patients with borderline performance status before surgery, as performance may be expected to decline further after surgery in some patients, making adjuvant chemotherapy harder to administer [6].

In short, this literature-based analysis suggests that NCT with protocols containing three drugs is more beneficial in stage 3 NSCLC than protocols with a lesser number of agents. An individual patient data meta-analysis testing this hypothesis is eagerly awaited; also, RCTs with third-generation drugs and three agents for NCT should be conducted separately for stage 3 disease.

## Conclusions

In this analysis of 14 RCTs of neoadjuvant chemotherapy before surgery, we show that in the setting of stage 3 NSCLC inclusion of 3 chemotherapy drugs in the treatment protocol is associated with less mortality. An individual patient data meta-analysis, as well as new RCTs, are needed to comment further on this issue.

## Competing interests

This work had no specific funding. The authors declare that they have no conflict of interest.

## Authors’ contributions

HB planned, designed and analyzed the study, collected data and wrote the manuscript, HA collected data, designed the study, SC helped writing the manuscript. All authors read and approved the final manuscript.

## Authors’ information

A complete list of the members of the Lung Cancer Committee is given at the end of the manuscript, in the acknowledgements section.
